# Author Correction: Bacterial nanotubes as a manifestation of cell death

**DOI:** 10.1038/s41467-020-19618-8

**Published:** 2020-11-03

**Authors:** Jiří Pospíšil, Dragana Vítovská, Olga Kofroňová, Katarína Muchová, Hana Šanderová, Martin Hubálek, Michaela Šiková, Martin Modrák, Oldřich Benada, Imrich Barák, Libor Krásný

**Affiliations:** 1 Laboratory of Microbial Genetics and Gene Expression, Institute of Microbiology of the Czech Academy of Sciences, 142 20 Prague 4, Czech Republic; 2 Laboratory of Molecular Structure Characterization, Institute of Microbiology of the Czech Academy of Sciences, 142 20 Prague 4, Czech Republic; 3 Department of Microbial Genetics, Institute of Molecular Biology, Slovak Academy of Sciences, 845 51 Bratislava, Slovakia; 4 Institute of Organic Chemistry and Biochemistry of the Czech Academy of Sciences, 160 00 Prague 6, Czech Republic; 5 Laboratory of Bioinformatics/Core Facility, Institute of Microbiology of the Czech Academy of Sciences, 142 20 Prague 4, Czech Republic

**Keywords:** Bacteria, Cellular microbiology, Microbial communities

Correction to: *Nature Communications* 10.1038/s41467-020-18800-2, published online 2 October 2020.

The original version of the Supplementary information associated with this Article omitted the Supplementary References. The HTML has been updated to include a corrected version of the Supplementary information.

