# Supplementary material for: Bacterial nanotubes as a manifestation of cell death
Source: Nat Commun. 2020 Oct 2;11:4963. doi: 10.1038/s41467-020-18800-2 (PMC7532143; doi:10.1038/s41467-020-18800-2)
Supplement: Supplementary file 1 — Supplementary Information [file 41467_2020_18800_MOESM1_ESM.pdf]

SUPPLEMENTARY INFORMATION

Bacterial nanotubes are a manifestation of cell death

Pospíšil *et al*.

Supplementary Figures 1-17

Supplementary Tables 1-4

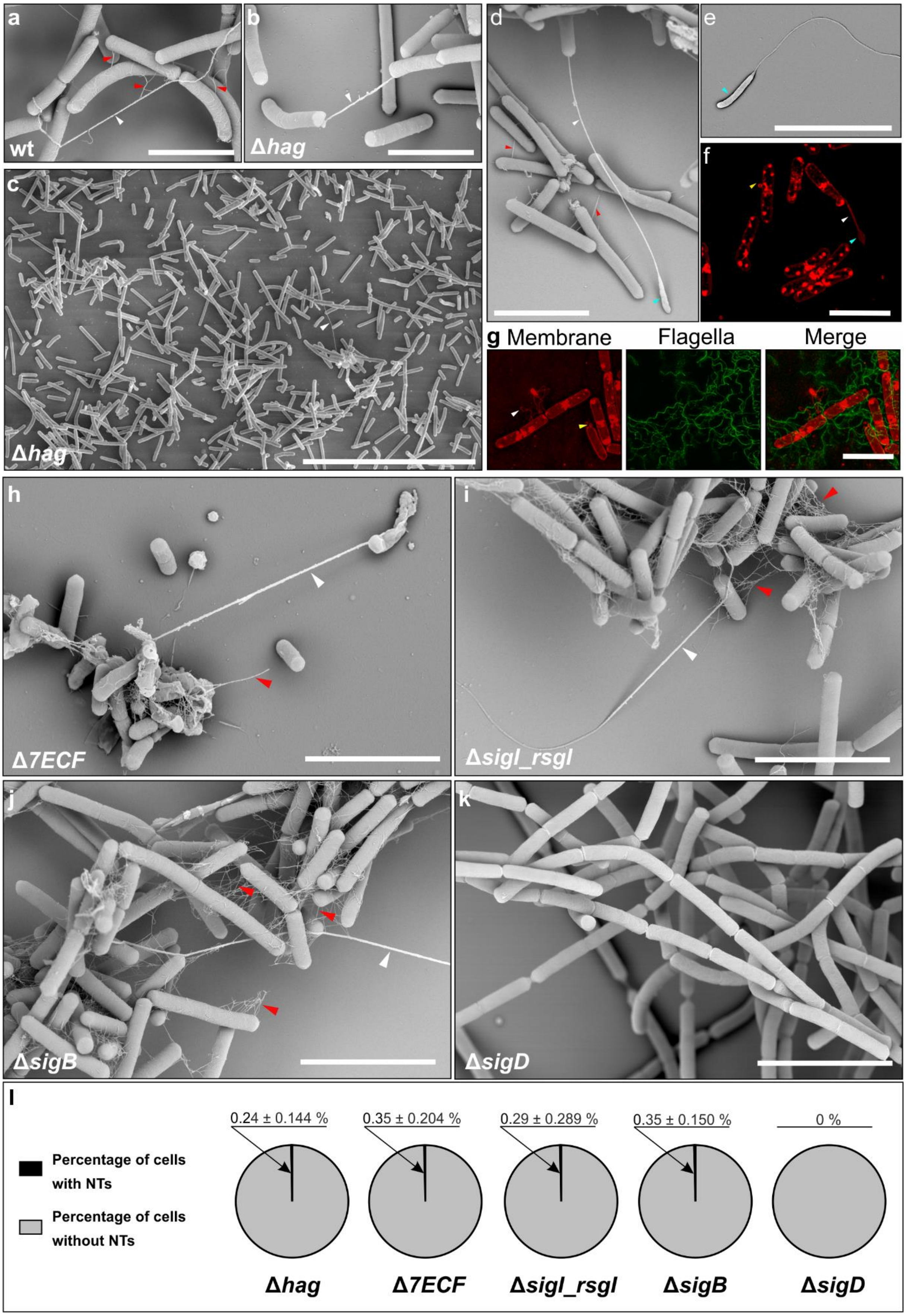

Supplementary Figure 1 | NT morphology, discrimination from flagella and identification of a sigma factor responsible for their formation.

SEM (Scanning Electron Microscopy) analysis of *B. subtilis* cells from the exponential phase of (**a**) wt (LK1432) and (**b**) Δ*hag* (LK1966) strains growing in liquid medium. White arrows indicate NTs; Red arrows indicate flagella. The scale bar=5 µm.

(**c**) A SEM image of the Δ*hag* strain showing the typical numbers of observed NTs for such image fields. The white arrow indicates the only NT in the field. Scale bar=30 µm.

(**d**-**e**) SEM images of the terminal structures of wt *B. subtilis* (LK1432) NTs. Scale bar=5 µm.

(**f**) A SIM image of a terminal structure of a wt *B. subtilis* NT. White arrows indicate NTs; Red arrows indicate flagella; Yellow arrows indicate membrane vesicles; Cyan arrows indicate terminal structures. Scale bar=5 µm.

(**g**) SIM microscopy of the hag^{T209C} (LK2052) strain containing a mutated *hag* gene[19]. The flagella are visualized using AF488-maleimide (green), membranes are stained by Nile red (red). White arrows indicate nanotubes; Yellow arrows indicate membrane vesicles. Scale bar=5 µm.

(**h**-**k**) SEM analysis of cells from exponential phase. Wt (LK1432) and deletion strains of various sigma factors (BSU2007 – Δ7ECF; LK2218 - Δ*sigB*; LK1550 - Δ*sigI*; LK1873 - Δ*sigD*) were imaged as described in Mat & Met. White arrows indicate nanotubes; Red arrows indicate flagella. Only the Δ*sigD* (LK1873) strain did not form NTs (it also did not form flagella). The scale bar=5 µm. All experiments (panels a-k) were conducted in at least three biological replicates (except for experiments in panel j, where two biological replicates were carried out).

(**l**) Quantification of NTs based on SEM analysis. Thick tubular structures (marked with white arrows in other panels) were counted as NTs. For each strain, ca 500 cells were analyzed for NT presence. The cell images used for the analysis were from three independent biological replicates, except for the Δ*sigB* strain where two biological replicas were used but the number of analyzed cells was 1,000. The pie chart represents 100%. Grey, cells without NTs; Black, cells with NTs (this percentage including ±SEM [Standard Error of the Mean] is indicated above each chart). The difference between wt and the mutant strains was not statistically significant. Nevertheless, the difference between wt and Δ*sigD* is highly significant $p<0.001$ when a different microscopic technique is used (Fig. 2c [wt] versus Supplementary Fig. 6b [Δ*sigD*]).

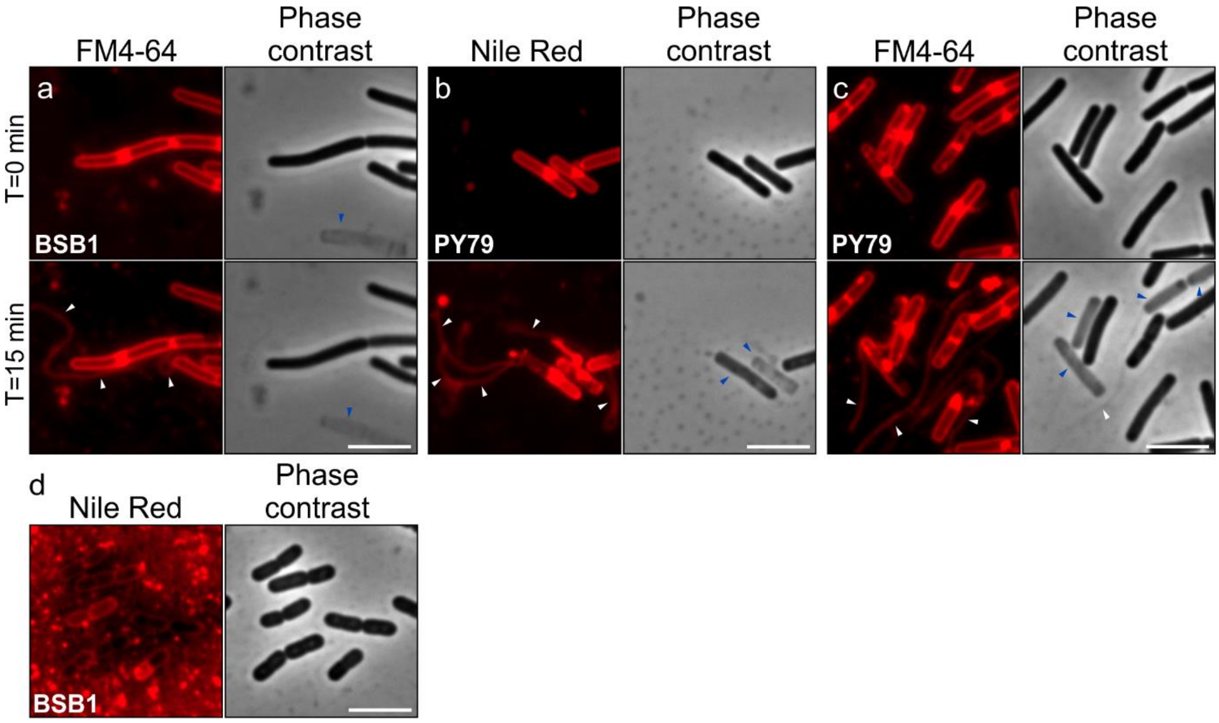

Supplementary Figure 2 | Comparison of the membrane staining of *B. subtilis* BSB1 and PY79 strains with Nile Red and FM4-64.

Fluorescence microscopy of exponentially grown *B. subtilis* BSB1 (LK1432) and PY79 (LK1629) strains. Bacteria were stained with Nile Red or FM4-64 (both in red) and samples were prepared by the P-GLG method. Pictures were taken at the indicated time points. White arrows indicate NTs; Blue arrows indicate ghost cells. Scale bar=5 µm. All experiments (panels a-d) were conducted in three biological replicates.

(**a**) The BSB1 strain stained with FM4-64.

(**b**) The PY79 strain stained with Nile Red.

(**c**) The PY79 strain stained with FM4-64.

(**d**) Example of incomplete staining of stationary phase *B. subtilis* cells with Nile Red.

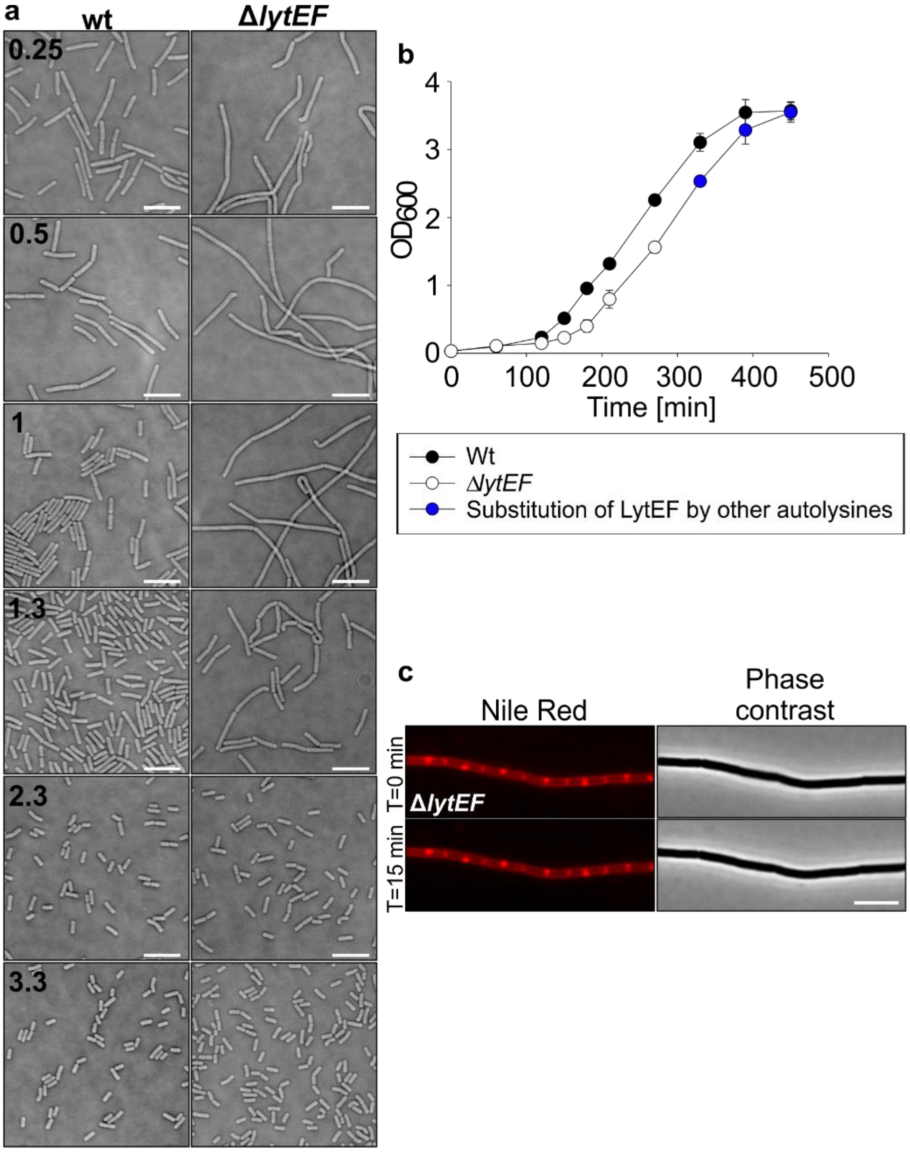

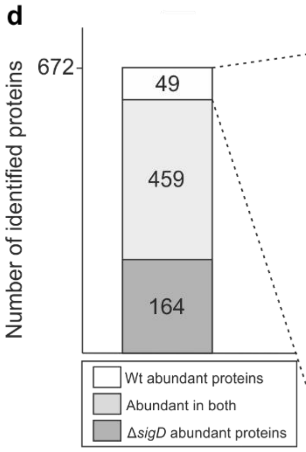

Wt abundant proteins	
**Functional group**	**Name**
**Motility and chemotaxis**	CheW*, FlgE*, FliF*, FliG*, FliL*, FliM*, FliY*, Hag$^{\Delta}$, HemAT$^{\Delta}$, McpA$^{\Delta}$, McpB$^{\Delta}$, McpC$^{\Delta}$, MotA$^{\Delta}$, MotB$^{\Delta}$, TlpB$^{\Delta}$, YfmS$^{\Delta}$, YoaH$^{\Delta}$, YvaQ$^{\Delta}$
**Cell wall degradation/turnover**	LytA*, LytB*, LytF$^{\Delta}$, XlyB
**SP-beta prophage**	YokF, YomK, YonS, YorG
**Lipid metabolism**	FabHA, FadE
**Carbon metabolism**	AcoC, BdhA, LutA, LutC
**Iron metabolism**	SufB
**Cell division**	DivIVA
**Resistance against oxidative and electrophile stress**	AhpF
**ATP synthesis**	SucD
**protein synthesis, modification and degradation**	CheA*, ClpP
**Proteins of unknown function**	CinA, YdjI, YlxF*, YpzJ, Yxkc$^{\Delta}$, YfmT$^{\Delta}$
**DNA condensation/segregation**	SftA
**Nucleotide metabolism**	RelA
**Regulation of gene expression**	SigA*, CheV$^{\Delta}$, CheY*

*Expression under the control of SigD and additional sigma factors.

$^{\Delta}$ Expression only under the control of SigD.

Supplementary Figure 3 | Morphology of the LytE and LytF autolysin deletion mutant in exponential phase and MS analysis of wt and Δ*sigD* membrane fractions.

(**a**) Wt *B. subtilis* (LK1432) and Δ*lytEF* (LK2290) strains were grown in liquid LB and cell morphology was observed by bright field microscopy at the indicated optical densities (OD_{600}) during culture growth. Scale bar=5 µm. The experiment was conducted in 3 biological replicates with similar results.

(**b**) Growth curves of the wt and Δ*lytEF* strains. Blue points indicate times when the functions of LytE and LytF are taken over by other autolysins. Data represent 3 biological replicates, shown as averages, the error bars indicate ±SEM [Standard Error of the Mean].

(**c**) Exponentially growing culture of the Δ*lytEF* strain was stained with Nile Red and prepared by the P-GLG method. Pictures were taken at the indicated time points. Scale bar=5 µm. Experiment was performed in 3 biological replicates with similar results.

(**d**) Protein mass spectrometry analysis of the wt (LK1432) and Δ*sigD* (LK1873) membrane fractions. The graph shows the number of genes sorted according to the membrane fractions in which they were found. The table lists those proteins that were enriched in the wt compared to Δ*sigD* (t-value ≥ 2, according to student's t-test). See Supplementary Tables 3 and 4 for the lists of proteins found in the other two fractions.

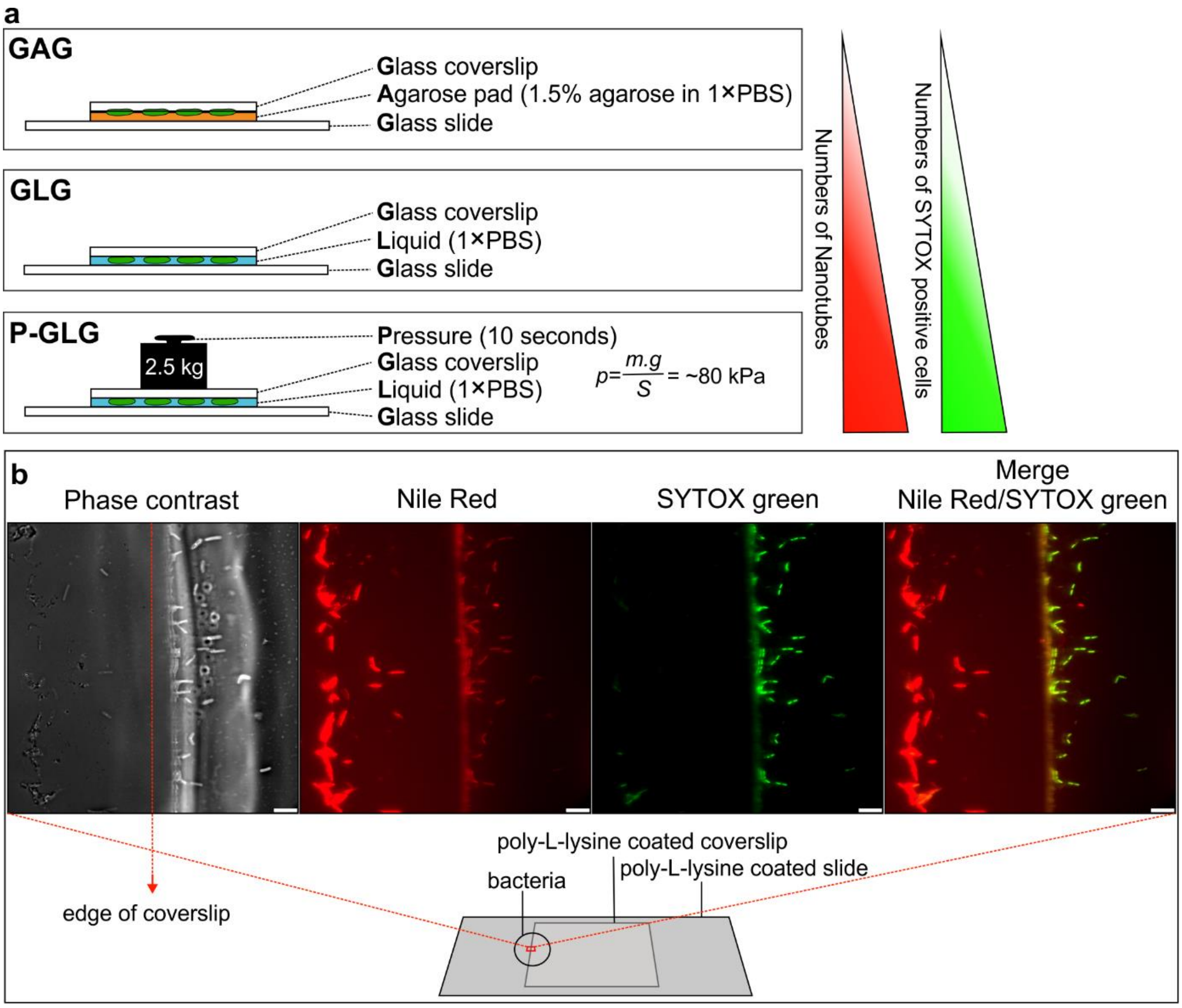

Supplementary Figure 4 | Different methods of sample preparation and death under the coverslip.

(**a**) Scheme of different methods of sample preparation. Red and green triangles indicate the trends in NT formation and the numbers of dying cells (monitored by SYTOX green).

(**b**) Exponentially grown wt (LK1432) cells were spotted on a poly-L-lysine coated glass slide and partially covered with a poly-L-lysine coverslip. Pressure was then applied on the coverslip as in the P-GLG method. The area around the edge of the coverslip was observed as indicated in the drawing. Membranes were stained with Nile Red (red) and cell death was determined by SYTOX green (green). Scale bar=5 µm. The experiment was conducted in 2 biological replicates with similar results.

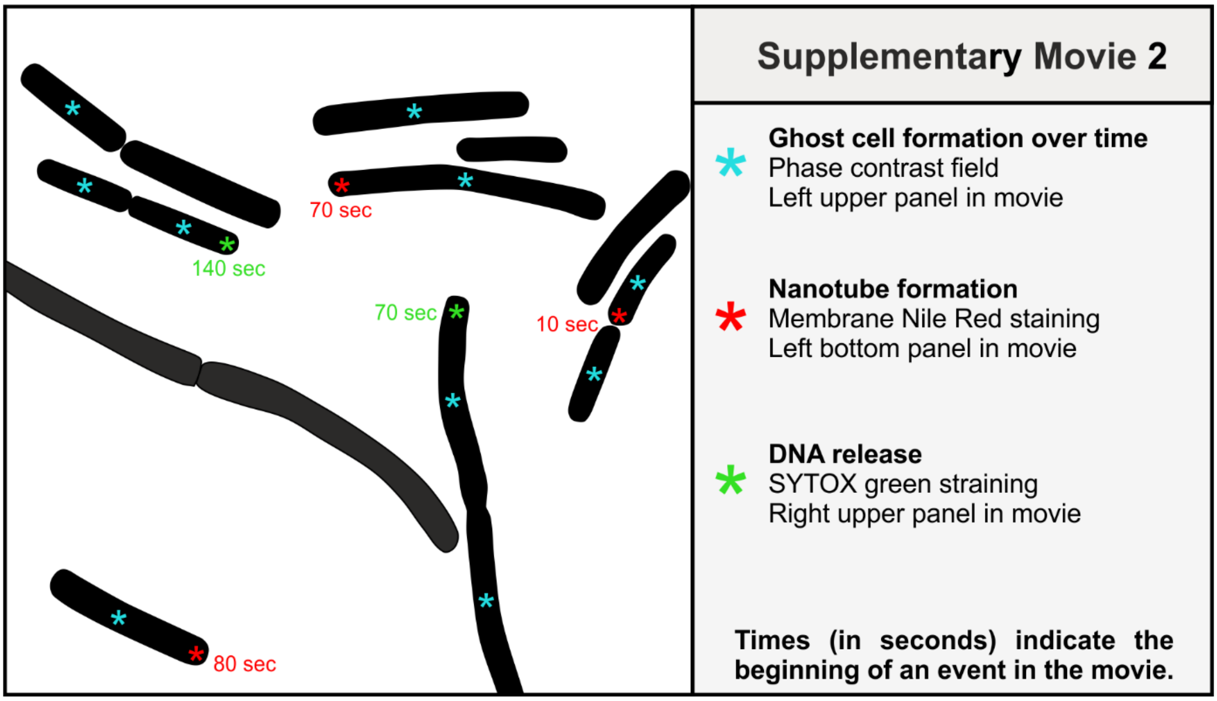

Supplementary Figure 5 | *B. subtilis* time-lapse fluorescence microscopy movie. Supplementary Movie 2 description.

This Supplementary Movie shows events occurring in dying cells under the P-GLG experimental setup. Colored asterisks indicate specific events (see legend next to the scheme). Seconds beside the asterisks indicate times at which these events begin.

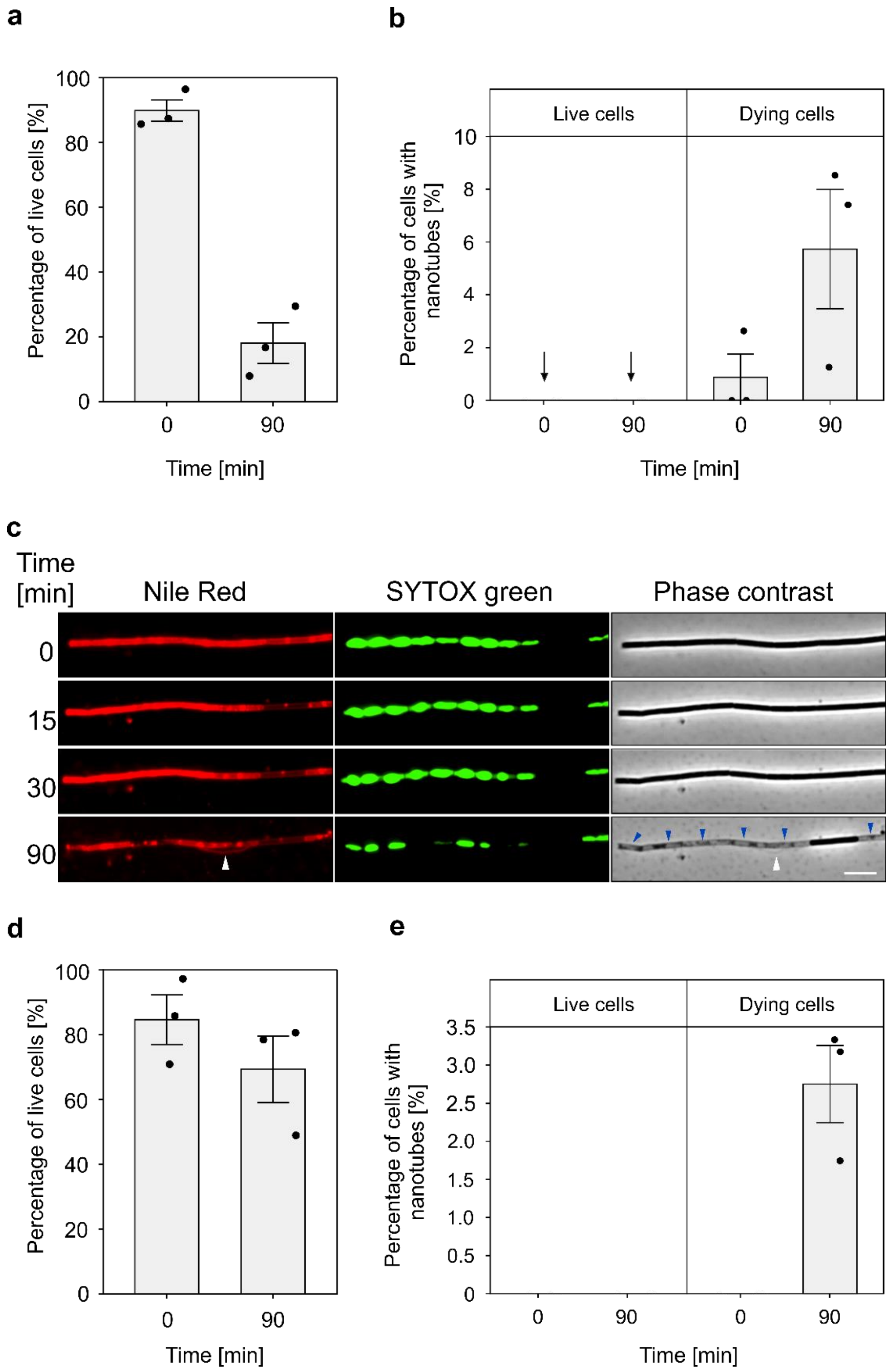

Supplementary Figure 6 | Delay in NT formation in the Δ*sigD* and Δ*lytEF* strains.

(**a**) Quantifications of live/dying Δ*sigD* (LK1873) cells were done based on the absence/presence of the SYTOX signal inside cells (sample was prepared by P-GLG method). The decrease in live cells numbers from t=0 to t = 90 is statistically significant ($p < 0.001$, GLM, two sided, $z = -24$, 95% CI OR = 0.02 – 0.03). 500 cells from 3 independent experiments were analyzed. The bars are averages and error bars ±SEM. The dots in this and other bar graphs represent individual experiments.

(**b**) Quantifications of the presence/absence of Δ*sigD* NTs were based on the Nile Red signal. The percentage of NTs is expressed relative to the number of dying (SYTOX positive) or live (SYTOX negative) cells, which was set as 100% (*e. g.* if out of 10% of dying cells, 20% formed NTs, then this would represent 2% of the total number of cells). 500 cells from 3 independent experiments were analyzed. The difference between the amounts of NTs formed by dying and live cells at t = 90 is statistically significant (p = 0.03, GLM, two sided, z = -2.2, 95% CI OR = 0.06 – 0.7). The bars are averages and error bars ±SEM. The vertical arrows in the graph indicate zero values.

(**c**) Exponentially grown *B. subtilis* Δ*lytEF* (LK2290) cells were stained with Nile Red (red), SYTOX green (green) and observed at the indicated time points (sample was prepared by the P-GLG method). The third column shows phase contrast images. White arrows indicate NTs. Blue arrows indicate ghost cells in the phase contrast fields. Scale bar=5 µm. Experiment was conducted in 3 biological replicates with similar results.

(**d**) Quantifications of live/dying Δ*lytEF* (LK2290) cells were done based on the absence/presence of the SYTOX signal inside cells (sample was prepared by P-GLG method). The decrease in live cells numbers from t = 0 to t = 90 is statistically significant (p < 0.001, GLM, two-sided, z = -7, 95% CI OR = 0.3 – 0.5). 500 cells from 3 independent experiments were analyzed. The bars are averages and error bars ±SEM.

(**e**) Quantification of the presence/absence of Δ*lytEF* NTs was based on the Nile Red signal. The percentage of NTs is expressed relative to the number of dying (SYTOX positive) or live (SYTOX negative) cells, which was set as 100% (e. g. out of the 10% of dying cells, 20% formed NTs – that is 2% of the total number of cells). 500 cells from 3 independent experiments were analyzed. The difference between the percentage of dying and live cells forming NTs at t = 90 is statistically significant (p = 0.007, GLM, two-sided, z = -2.6, 95% CI OR = 1.7 - 27). The bars are averages and error bars ±SEM. The vertical arrows in the graph indicate zero values.

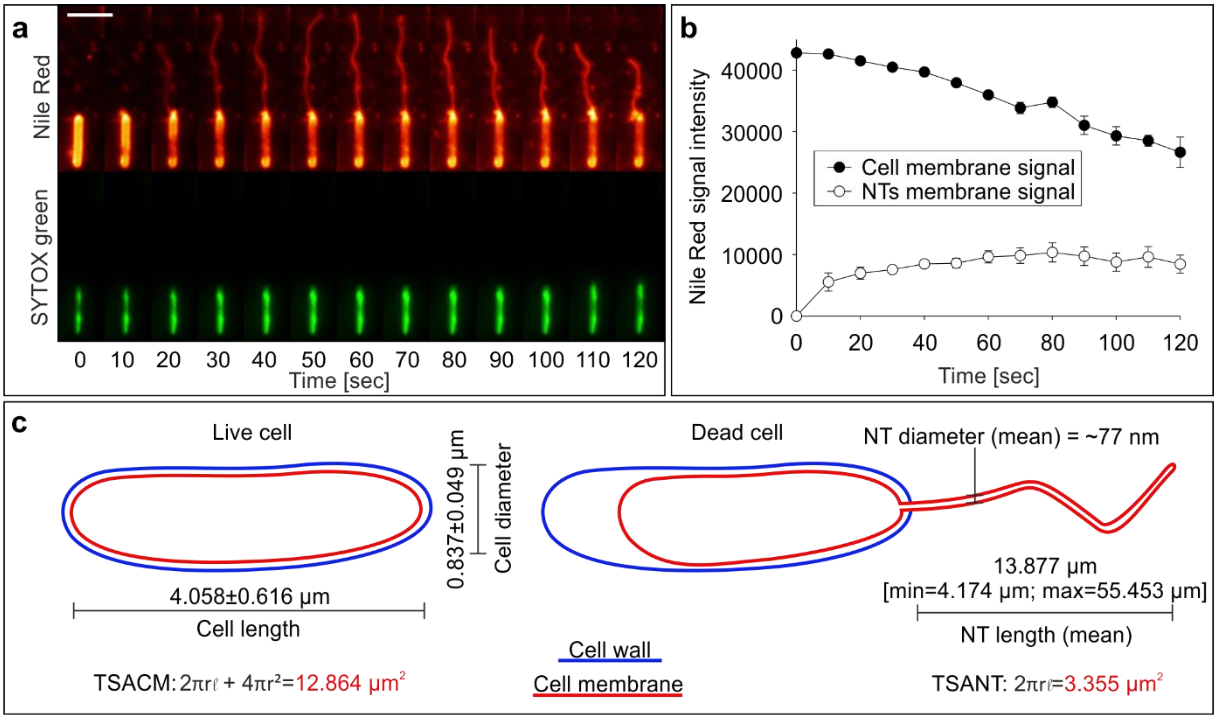

Supplementary Figure 7 | NTs are formed using the existing plasma membrane.

(**a**) Wt (LK1432) cells were prepared as in Figure 3 (main text). The primary data show time lapse (10 sec intervals) of a representative cell. The upper panel shows membrane stain Nile Red (false colored glow), the lower panel shows SYTOX (green). Note the decrease in the Nile red intensity in the cell area, concomitant with NT extrusion. Scale bar=5 µm. Experiment was conducted in 3 biological replicates with similar results.

(**b**) Quantification of the Nile Red signal in the cell area (closed circles) and in the NT area (open circles). After bleaching and background-subtraction corrections, the Nile red signal was quantified from 5 different NT-forming cells from one representative experiment. The error bars indicate ±SEM.

(**c**) Calculations of the **T**otal **S**urface **A**rea of the **C**ell **M**embrane (TSACM) and **T**otal **S**urface **A**rea of the **NT** (TSANT). After cell death, the NT is built from the cell's membrane. The length (l) and diameter (2r) of the Nile Red-stained cells (the membrane envelope dimensions) were measured in SIM images (from 100 exponentially growing cells). NT lengths were measured from fluorescence microscopy images. NT diameters were measured from SEM micrographs (10 NTs) because fluorescence microscopy does not have sufficient resolution for this and NT movement in SIM images may cause this parameter to be overestimated.

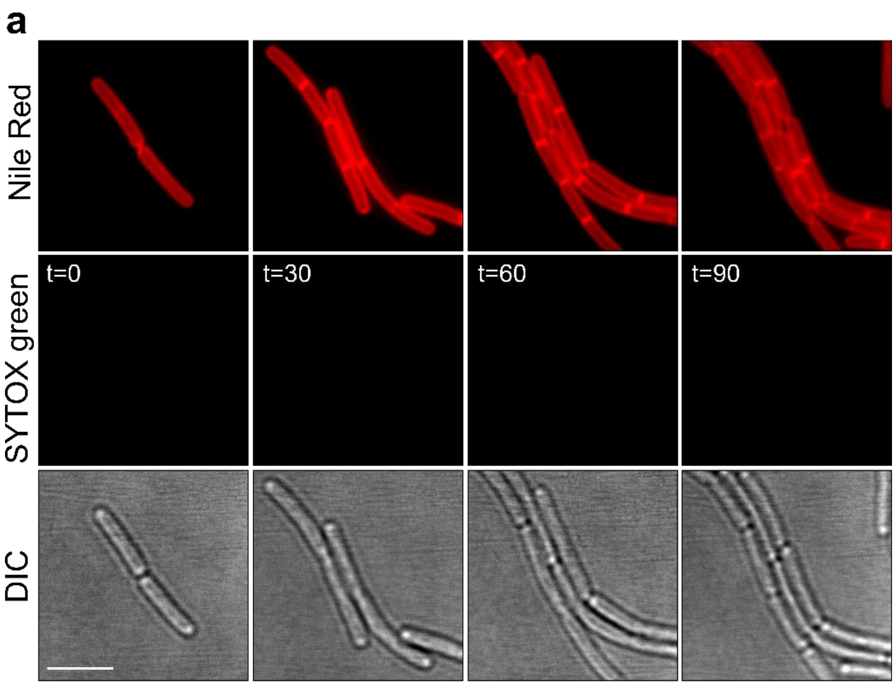

Time [min]	0	30	60	90
Live/Dying cells	159/0	262/7	557/9	1245/23
Live/Dying cells NTs	0/0	0/0	0/0	0/0

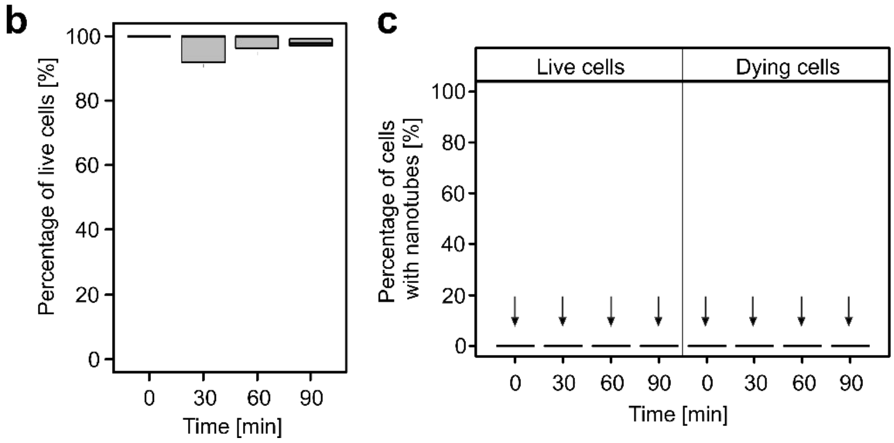

Supplementary Figure 8 | Time-lapse images of *B. subtilis* cells grown in frames on LB agarose without antibiotic supplementation at 37 °C.

(**a**) Exponentially growing wt cells (LK1432) were stained with Nile Red (red) and spotted on LB agar in gene frames (*i.e.* 'no pressure' conditions) containing SYTOX green (green). Pictures were taken every 30 minutes and the numbers of live/dying cells and the numbers of NTs emerging from live/dying cells were counted and are shown below the images (summary of 3 independent biological repeats). Scale bar=5 µm.

(**b**) Box plot shows the percentage of live cells at indicated time points. The differences are not statistically significant. The data are from 3 biological replicates.
Box plots were plotted with default R setting: center line = median; vertical size of the boxes = interquartile range (IQR); box limits = upper and lower quartiles; whiskers = 1.5x interquartile range (1.5×IQR).

(**c**) Quantification of the presence/absence of NTs at the indicated time points. Quantification of NTs was based on the Nile Red signal. The values are in all cases 0 as no NTs were detected with this technique. The vertical arrows in the graph indicate zero values in all replicates. Data represent 3 biological replicates. Box plots are defined as in chart b.

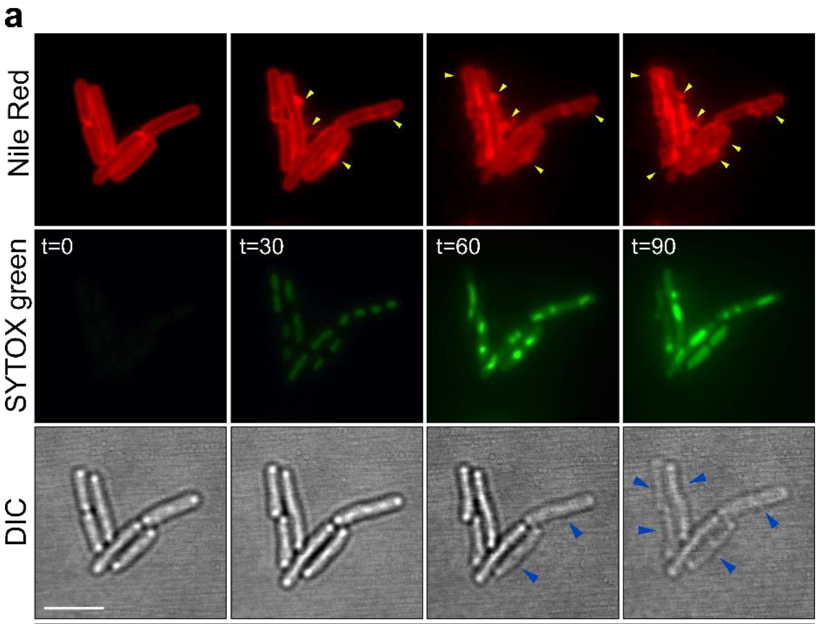

Time [min]	0	30	60	90
Live/Dying cells	605/9	0/614	0/614	0/614
Live/Dying cells NTs	0/0	0/4	0/18	0/22

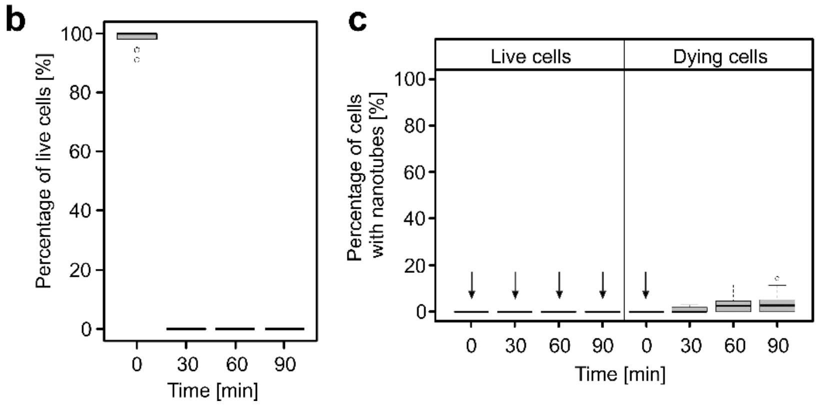

Supplementary Figure 9 | Time-lapse images of *B. subtilis* cells grown in frames on LB agarose supplemented with ampicillin at 37 °C.

(**a**) Exponentially growing wt cells (LK1432) were stained with Nile Red (red) and spotted on LB agar containing ampicillin (500 µg/ml) and SYTOX green (green) in gene frames (*i.e.* 'no pressure' conditions). Pictures were taken every 30 minutes and the numbers of live/dying cells and the numbers of NTs emerging from live/dying cells were counted and are shown below the images (summary of 3 independent biological repeats). Yellow arrows indicate membrane blebs and vesicles. Dark blue arrows indicate dying cells in DIC images. Scale bar=5 µm. The data are from 3 biological replicates.

(**b**) Box plot shows the percentage of live cells at indicated time points, decreasing over time ($p < 0.001$, GLM, two-sided, $z = -24$, 95% CI OR = 0.01 – 0.03). Box plots were plotted with default R setting: center line = median; vertical size of the boxes = interquartile range (IQR); box limits = upper and lower quartiles; whiskers = 1.5x interquartile range (1.5×IQR); points (open circles) = outliers.

(**c**) Quantification of the presence/absence of NTs at the indicated time points. Quantification of NTs was based on the Nile Red signal. The percentage of NTs is expressed relative to the number of dying (SYTOX positive) or live (SYTOX negative) cells, which was set as 100% (e. g. out of the 10% of dying cells, 20% formed NTs – that is 2% of the total number of cells). The percentage of NTs of dying cells increases over time ($p = 0.001$, GLM, two-sided, $z = 3.2$, 95% CI OR = 1.3 – 2.8). The vertical arrows in the graph indicate zero values in all replicates. Data represent 3 biological replicates. Box plots are defined as in chart b.

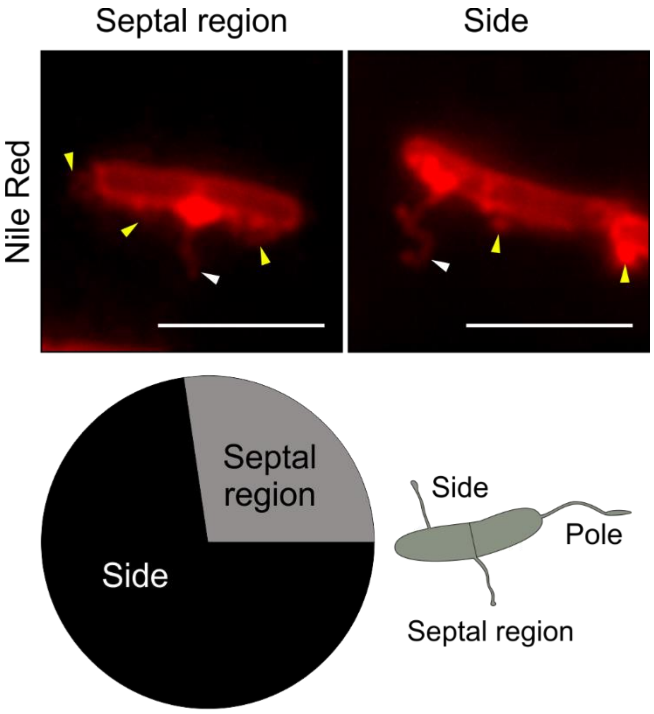

Supplementary Figure 10 | Quantification of the sites of NT origin after ampicillin treatment.

Dying wt cells (LK1432) containing NTs were analyzed with respect to the position on NT attachment (polar, septal, from sides). The cells were stained with Nile Red to visualize membranous structures. White arrows indicate NTs. Yellow arrows indicate membrane blebs. Scale bar=5 µm. A total of 22 cells were analyzed, selected from 3 biological replicates, with similar results in each replicate. The overall trend is shown in the pie chart: No NTs emanating from the cell poles were found. NTs originating from the sides prevailed (16 NTs) over NTs formed from septa (6 NTs).

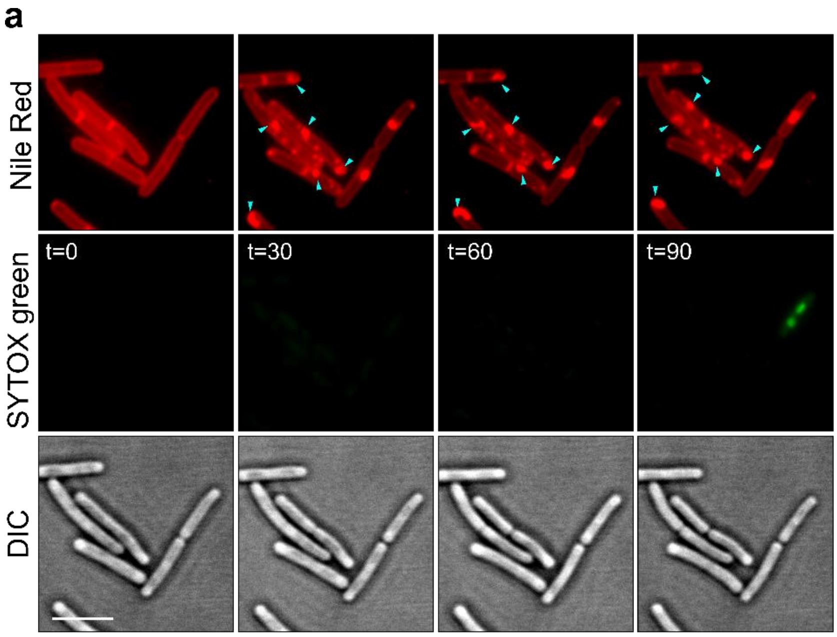

Time [min]	0	30	60	90
Live/Dying cells	629/0	622/7	605/24	539/90
Live/Dying cells NTs	0/0	0/0	0/0	0/1

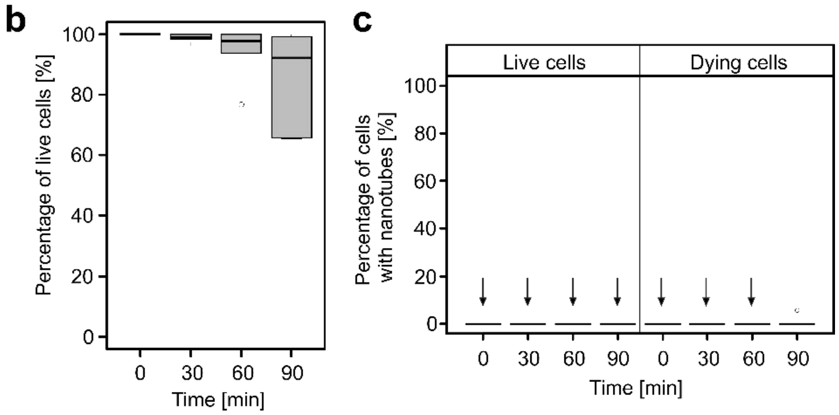

Supplementary Figure 11 | Time-lapse images of *B. subtilis* cells grown in frames on LB agarose supplemented with chloramphenicol at 37 °C.

(**a**) Exponentially growing wt cells (LK1432) were stained with Nile Red (red) and spotted on LB agar containing chloramphenicol (5 µg/ml) and SYTOX green (green) in gene frames (*i.e.* 'no pressure' conditions). Pictures were taken every 30 minutes and the numbers of live/dying cells and the numbers of NTs emerging from live/dying cells were counted and are shown below the images (summary of 3 independent biological repeats). Light blue arrows indicate dying cells in DIC images. Scale bar=5 µm.

(**b**) Box plot shows the percentage of live cells at indicated time points, decreasing over time ($p < 0.001$, GLM, two-sided, $z = -9$, 95% CI OR = 0.16 – 0.30). Box plots were plotted with default R setting: center line = median; vertical size of the boxes = interquartile range (IQR); box limits = upper and lower quartiles; whiskers = 1.5x interquartile range (1.5×IQR); points (open circles) = outliers. The data are from 3 independent biological replicates.

(**c**) Quantification of the presence/absence of NTs at the indicated time points. Quantification of NTs was based on the Nile Red signal. The values are in all but one case 0 as no NTs were detected at these time points. The only exception is the last time point (dying cells) where a single NT was detected. The vertical arrows in the graph indicate zero values in all replicates. Data represent 3 independent biological replicates. Box plots are defined as in chart b.

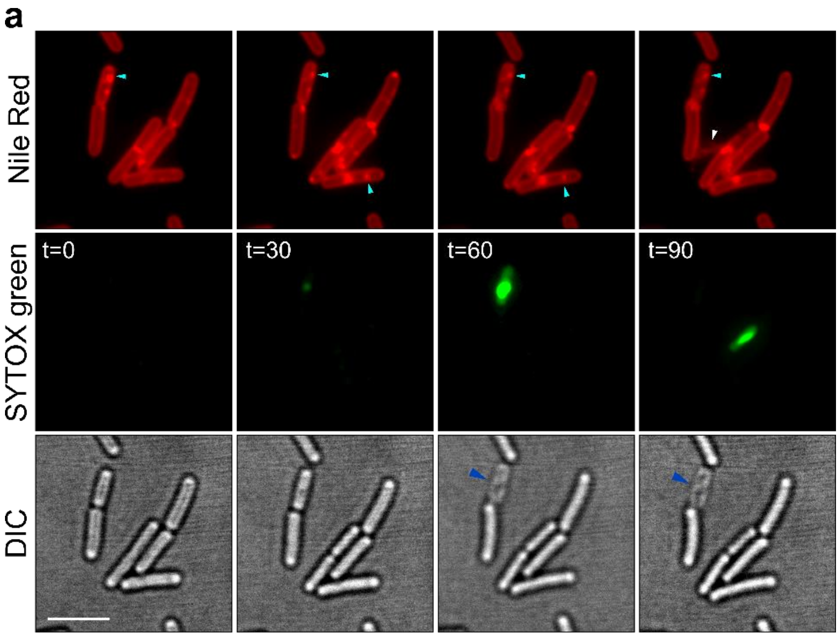

Time [min]	0	30	60	90
Live/Dying cells	749/0	739/10	729/20	693/56
Live/Dying cells NTs	0/0	0/0	0/0	0/1

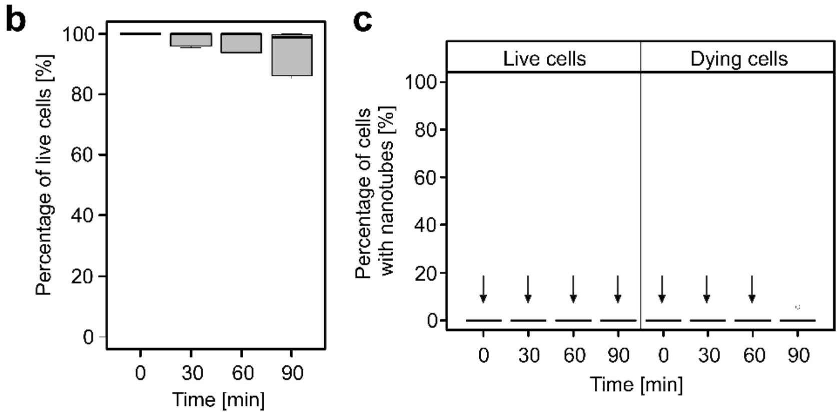

Supplementary Figure 12 | Time-lapse images of *B. subtilis* cells grown in frames on LB agarose supplemented with rifampicin at 37 °C.

(**a**) Exponentially growing wt cells (LK1432) were stained with Nile Red (red) and spotted on LB agar containing rifampicin (50 µg/ml) and SYTOX green (green) in gene frames (*i.e.* 'no pressure' conditions). Pictures were taken every 30 minutes and the numbers of live/dying cells and the numbers of NTs emerging from live/dying cells were counted and are shown below the images (summary of 3 independent biological repeats). Light blue arrows indicate impaired membranes, white arrow indicates nanotube, dark blue arrows indicate dying cells in DIC images. Scale bar=5 µm.

(**b**) Box plot shows the percentage of live cells at indicated time points, decreasing over time ($p < 0.001$, GLM, two-sided, $z = -7$, 95% CI OR = 0.24 – 0.43). The data are from 3 independent biological replicates. Box plots were plotted with default R setting: center line = median; vertical size of the boxes = interquartile range (IQR); box limits = upper and lower quartiles; whiskers = 1.5x interquartile range (1.5×IQR).

(**c**) Quantification of the presence/absence of NTs at the indicated time points. Quantification of NTs was based on the Nile Red signal. Quantification of NTs was based on the Nile Red signal. The values are in all but one case 0 as no NTs were detected at these time points. The only exception is the last time point (dying cells) where a single NT was detected. The vertical arrows in the graph indicate zero values in all replicates. Data represent 3 independent biological replicates. Box plots are defined as in chart b; the point (open circle) = outlier.

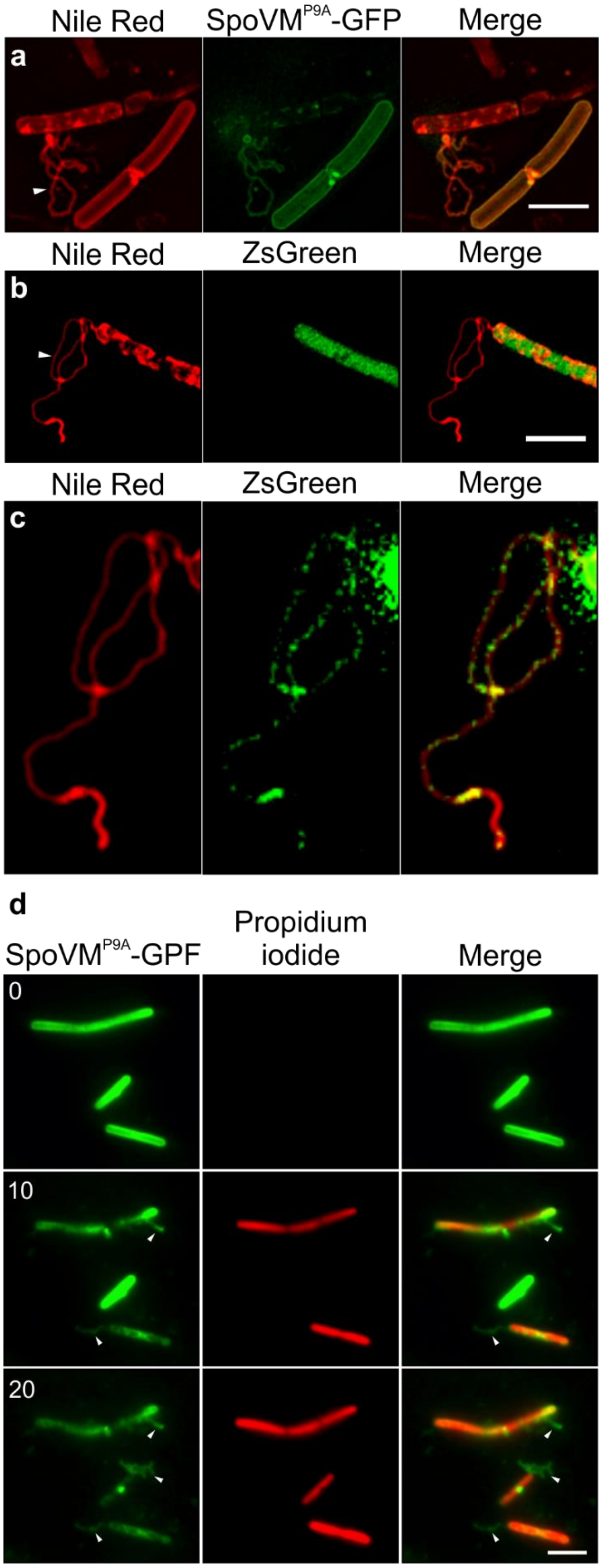

Supplementary Figure 13 | SpoVM^{P9A}-GFP and GFP localize to NTs.

(**a-c**) Exponentially growing *B. subtilis* cells prepared by the P-GLG method and observed by SIM. Membranes were stained with Nile Red (red), ZsGreen and SpoVM^{P9A} (green). White arrows indicate NTs. Scale bar=2.5 µm.

(**a**) Strain LK2525 harboring a mutated variant of the SpoVM protein (SpoVM^{P9A}) that binds indiscriminately to cell membranes[56].

(**b**) Strain LK2375 producing free cytosolic ZsGreen (green fluorescence protein[57]. The brightness and contrast in panels **a** and **b** are identical. At these settings, free GFP was not detected in the NT.

(**c**) Brightness was adjusted relative to panel **b** to visualize ZsGreen in the NT.

(**d**) Fluorescence microscopy. The SpoVM^{P9A}-GFP (LK2525) strain was grown in the presence of 1mM IPTG into exponential phase. Cells were harvested and re-suspended in 1× PBS containing Propidium iodide. The sample was prepared by the GLG method. Pictures were taken after 0, 10 and 20 minutes. Propidium iodide (red) revealed that the bacterial population began to die after 10 minutes. Dying cells formed tubular structures (white arrows) that were full of the SpoVM^{P9A}-GFP (green) membrane protein. Interestingly, we again observed SpoVM^{P9A}-GFP signal decay in cell during NT production, which suggests that SpoVM^{P9A}-GFP is extruded into the extracellular space together with (bound to) the membrane. Scale bar=5 µm.

Experiments in panels a-d were conducted in 3 biological replicates with similar results.

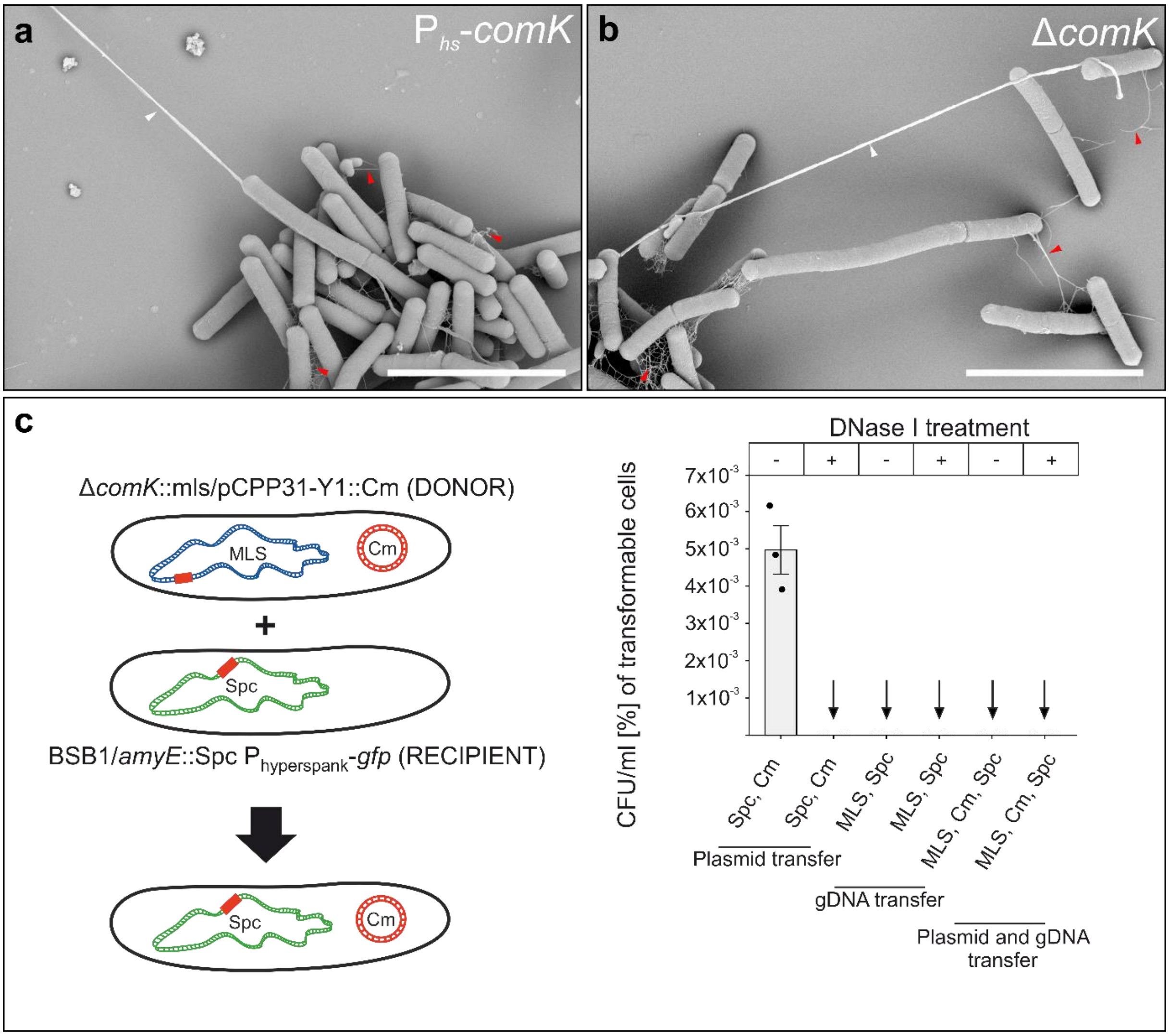

Supplementary Figure 14 | Distinguishing plasmid transfer from gDNA transfer.

(**a**-**b**) A SEM analysis of $P_{hyperspank}$-*comK* and Δ*comK* exponentially growing cells reveal that NTs formed by these strains were the same as those formed by wt. White arrows indicate nanotubes; Red arrows indicate flagella. The scale bar=5 µm. The experiment was conducted in 2 biological replicates with similar results.

(**c**) The donor strain (LK2392, Δ*comK*::mls/pCPP31-Y1::cm) was cocultivated with recipient strain (LK2398, *amyE*::Spc $P_{hyperspank}$-*gfp*) for 4 hours in LB without antibiotics. Double-resistant colonies were selected on LB agar plates containing appropriate antibiotics. We did not obtain any colonies on LB supplemented with MLS+Cm+ Spc or MLS+Spc. Double resistant colonies were obtained only on LB with Spc+Cm, which suggests non-conjugative plasmid transfer but not gDNA transfer. Importantly, the DNA transfer was abolished by DNase I treatment. The difference between Spc+Cm in the DNase- condition and all other conditions is statistically significant ($p < 0.001$). The bars are averages from three biological replicates and error bars show ±SEM.

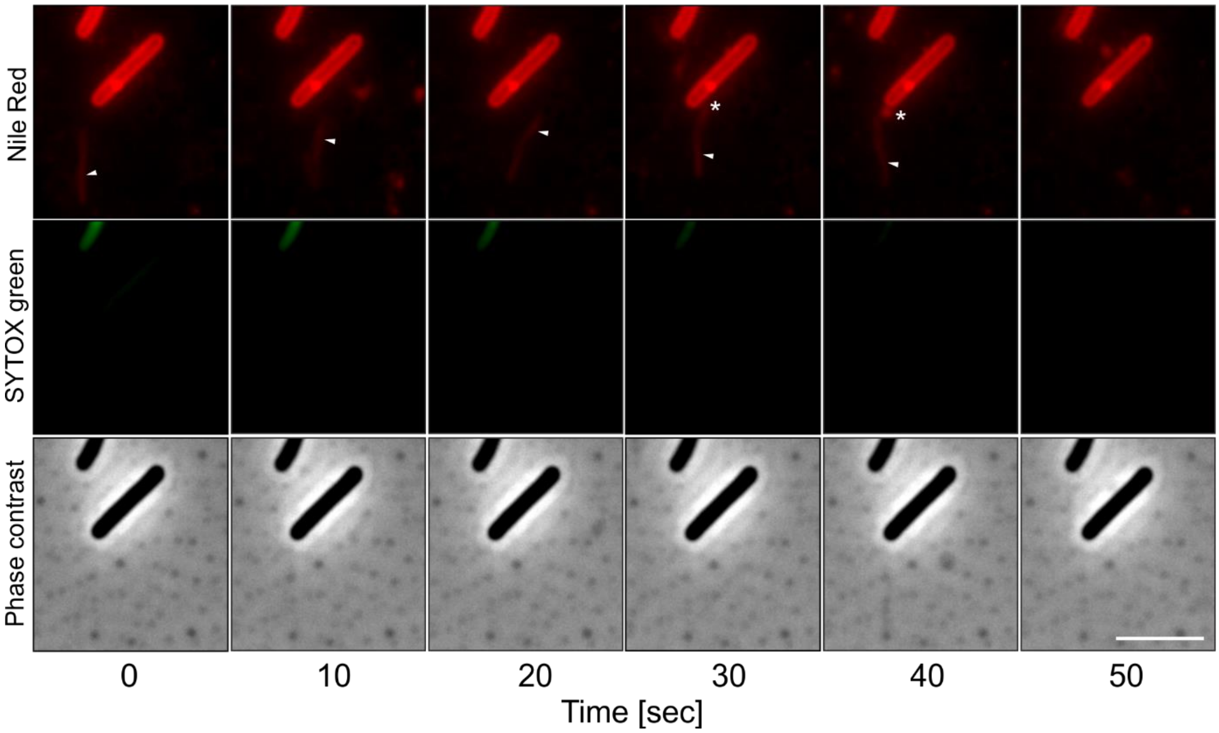

Supplementary Figure 15 | Nanotubes ‘connected’ to a living cell.

Time-lapse images of wt *B. subtilis* (LK1432) prepared by the P-GLG method. In some cases, after NT formation tubules are torn apart and dragged around by the capillary forces within the sample liquid. This NT fragment can be captured by a living cell, creating the illusion of an NT extruded from a living cell (white asterisk). The white arrows indicate the moving NT. Scale bar=5 µm. Experiment was conducted in at least 3 biological replicates with similar results.

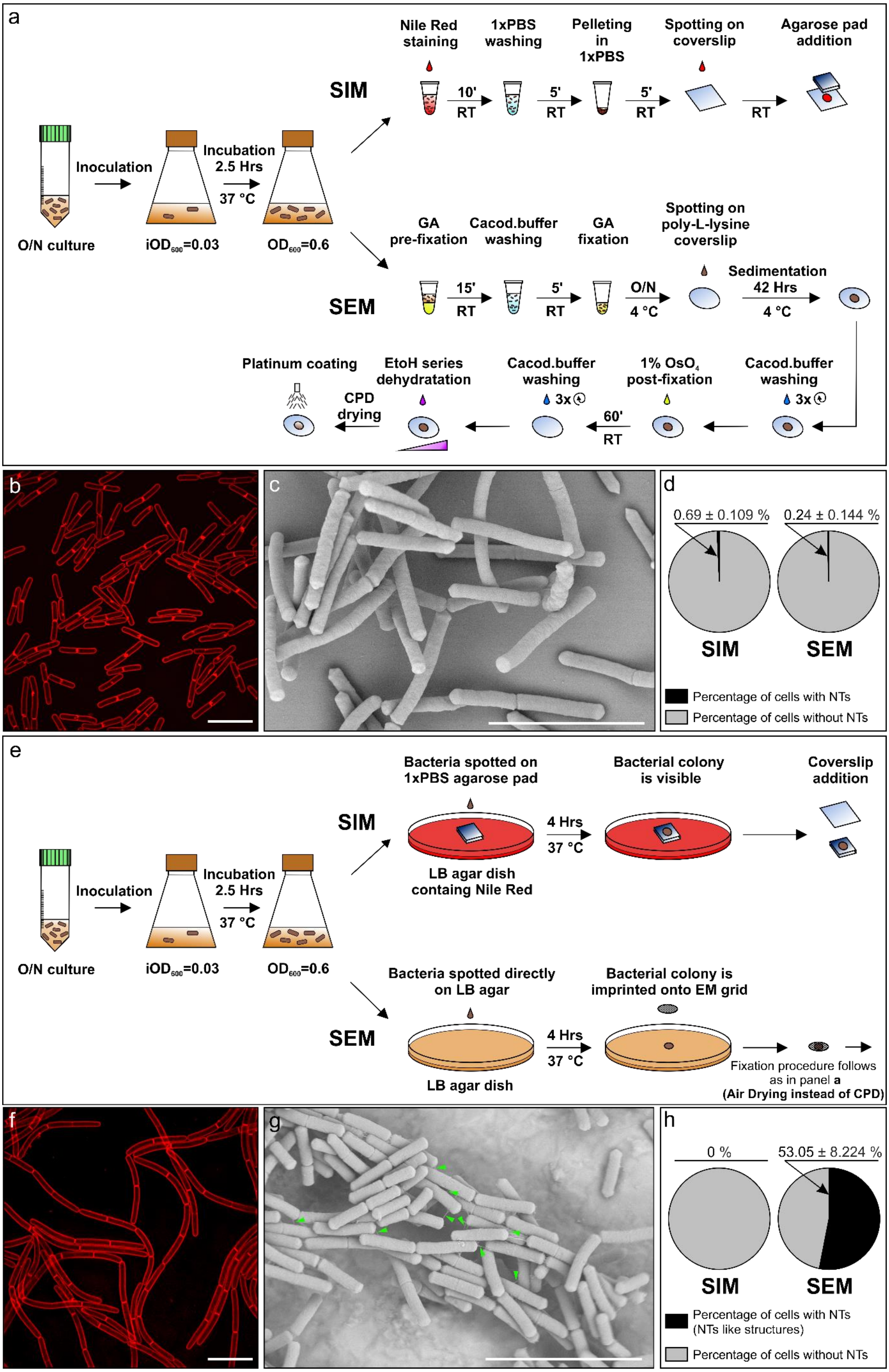
a
SIM
Nile Red staining
1xPBS washing
Pelleting in 1xPBS
Spotting on coverslip
Agarose pad addition
10' RT
5' RT
5' RT
RT
Inoculation
Incubation 2.5 Hrs 37 °C
O/N culture
iOD600=0.03
OD600=0.6
SEM
GA pre-fixation
Cacod.buffer washing
GA fixation
Spotting on poly-L-lysine coverslip
Sedimentation 42 Hrs 4 °C
15' RT
5' RT
O/N 4 °C
Platinum coating
EtoH series dehydratation
Cacod.buffer washing
1% OsO4 post-fixation
Cacod.buffer washing
CPD drying
3x
60' RT
b
c
d
0.69 ± 0.109 %
0.24 ± 0.144 %
SIM
SEM
Percentage of cells with NTs
Percentage of cells without NTs
e
Bacteria spotted on 1xPBS agarose pad
Bacterial colony is visible
Coverslip addition
4 Hrs 37 °C
LB agar dish containg Nile Red
Bacteria spotted directly on LB agar
Bacterial colony is imprinted onto EM grid
LB agar dish
Fixation procedure follows as in panel a (Air Drying instead of CPD)
f
g
h
0 %
53.05 ± 8.224 %
Percentage of cells with NTs (NTs like structures)
Percentage of cells without NTs

Supplementary Figure 16 | Sample preparation of bacteria grown in liquid media or on solid media.

(**a**) Scheme of sample preparation of bacteria grown in liquid media. Bacteria were inoculated from glycerine stocks into 10 ml of LB media with appropriate antibiotics and grown overnight (O/N). Next day, bacteria were inoculated at **i**nitial **O**ptical **D**ensity 0.03 (iOD_{600}) into 10 ml of fresh LB media without any antibiotic. The cultures were grown until mid-logarithmic phase (OD_{600}=0.6). For **SIM**, 1 ml of the bacterial culture was withdrawn and Nile Red was added at a final concentration of 10 µg/ml. After 10 minutes of incubation at room temperature (RT), bacteria were pelleted by centrifugation (6,000× g, 5 min at RT) and washed once with 1× PBS. Subsequently, bacteria were pelleted again a resuspended in 50 µl of 1× PBS. 3 µl of cell suspension were spotted on an uncoated coverslip and covered with a thin (ca 3 mm) 1× PBS agarose pad. The coverslip was placed into a holder and the sample was observed using DeltaVision OMX™. Alternatively - for **SEM** - cells were grown in the same way but immediately fixed. 0.5 ml of exponentially growing cells was added to 0.5 ml of 6% glutaraldehyde (GA pre-fixation). After 15 minutes of incubation at RT, bacteria were pelleted (6,000× g, 5 min at RT) and washed once with cacodylate buffer. Then, the sample was pelleted again, resuspended in 0.5 ml of 3% glutaraldehyde (GA fixation) and kept at 4 °C, O/N. Next day, the sample was spotted on a poly-L-lysine-treated circular glass coverslip and sedimented at 4°C in a Petri-dish moist chamber for 42 h. The cells attached to the coverslip were washed three times with the cacodylate buffer and postfixed in 1% OsO_4 for one hour at room temperature and subsequently washed three times with the cacodylate buffer. The coverslips were dehydrated through a graded ethanol series (25%, 50%, 75%, 90%, 96%, 100% and 100%) followed by absolute acetone and critical point-dried (CPD) in a K850 Critical Point Dryer. The dried samples were sputter-coated with 3 nm of platinum. The sample was observed using a FEI Nova NanoSEM scanning electron microscope.

(**b**) Membrane stained planktonic cells display few, if any, NTs. This large SIM field of Nile Red stained wt *B. subtilis* (LK1432) represents a typical picture. No NTs are visible in this field. Scale bar=5 µm. For examples of NTs that were identified by SIM see Supplementary Data Fig. 1f-g and Fig. 13. The experiment was conducted in at least 3 biological replicates with similar results.

(**c**) Planktonic culture of *B. subtilis* analyzed by SEM. Δ*hag* (LK1966, non-flagellated strain) cells were used to simplify interpretation. As in SIM, few, if any, tubular structure were detected. The picture represents a typical SEM field and contains no tubular structures. In rare cases, however, tubular structures were detected. Scale bar=5 µm. To view a larger field (in terms of cells) that also contains a single NT, see Supplementary Data Fig. 1c. The experiment was conducted in at least 3 biological replicates with similar results.

(**d**) Quantification of NTs based on SIM and SEM methods. For each method, ca 1,000 cells (from three independent replicates) were analyzed for NT presence. For SIM, NTs were detected based on the Nile Red fluorescence signal and thus only membranous tubular structures were strictly quantified. For SEM, “thick” (ca 77 nm – see Supplementary Data Fig. 6c) tubular structures (marked with white arrows in SEM figures) were counted as NTs. The pie chart represents 100%. Grey, cells without NTs; Black, cells with NTs (this percentage including ±SEM is indicated above each chart; the difference between the percentages is not statistically significant).

(**e**) Scheme of sample preparation of bacteria grown on solid media. Bacteria were inoculated from glycerine stocks into 10 ml of LB media with appropriate antibiotics and grown O/N. Next day, bacteria were inoculated at initial optical density 0.03 (iOD_{600}) into 10 ml of fresh LB without any antibiotics. Then, the culture was grown until mid-logarithmic phase (OD_{600}=0.6). For SIM, an exponential culture was 10x diluted in 1× PBS and 2 µl were spotted on a 1× PBS agarose pad lying on LB agar containing Nile Red (final conc. 20 µg/ml). Cells were then incubated at 37 °C for 4 hours in dark. Subsequently, the agarose pad containing the bacterial colony (that grew from the spotted culture) was removed from the dish and covered with an uncoated coverslip. The coverslip was placed into a holder and the sample was observed using DeltaVision OMX™. Alternatively - for SEM - cells were grown in the same way but bacteria were spotted directly on LB agar (without Nile Red). After four hours, a bacterial colony was imprinted onto a Electron microscopy (EM) grid. The following fixation procedure was identical as in panel a, except for the drying technique (air drying was used instead of CPD drying).

(**f**) SIM analysis of wt *B. subtilis* (LK1432) grown on solid media and stained by Nile Red. No NTs were detected. Scale bar=5 µm. The experiment was conducted in at least 3 biological replicates with similar results.

(**g**) SEM analysis of *B. subtilis* (LK1966, non-flagellated strain) grown on solid media. Numerous structures were identified between cells (green arrows). However, these structures were not identified by membrane staining (SIM) and thus they are unlikely to be made of membranes. Scale bar=5 µm. The experiment was conducted in at least 3 biological replicates with similar results.

(**h**) Quantification of NTs (or NT-like structures) emerging from cells cultured on solid medium and observed with SIM and SEM methods. For each method, ca 300 cells (from three independent replicates) were analyzed for NT presence. For SIM, no NTs were detected based on Nile Red fluorescence signal. For SEM, tubular filamentous structures were quantified (marked with green arrows in panel g). We stress that all these structures are unlikely to be NTs as this method is unable to distinguish between tubular structures of different origin (hence the term 'NT-like'). The pie chart represents 100%. Grey, cells without NTs; Black, cells with NTs/NT-like structures (this percentage including ±SEM is indicated above each chart; the difference between the SIM and SEM results is statistically significant [$p < 0.001$, GLM, two-sided, $z = 8$, 95% CI OR = 47 - 516]).

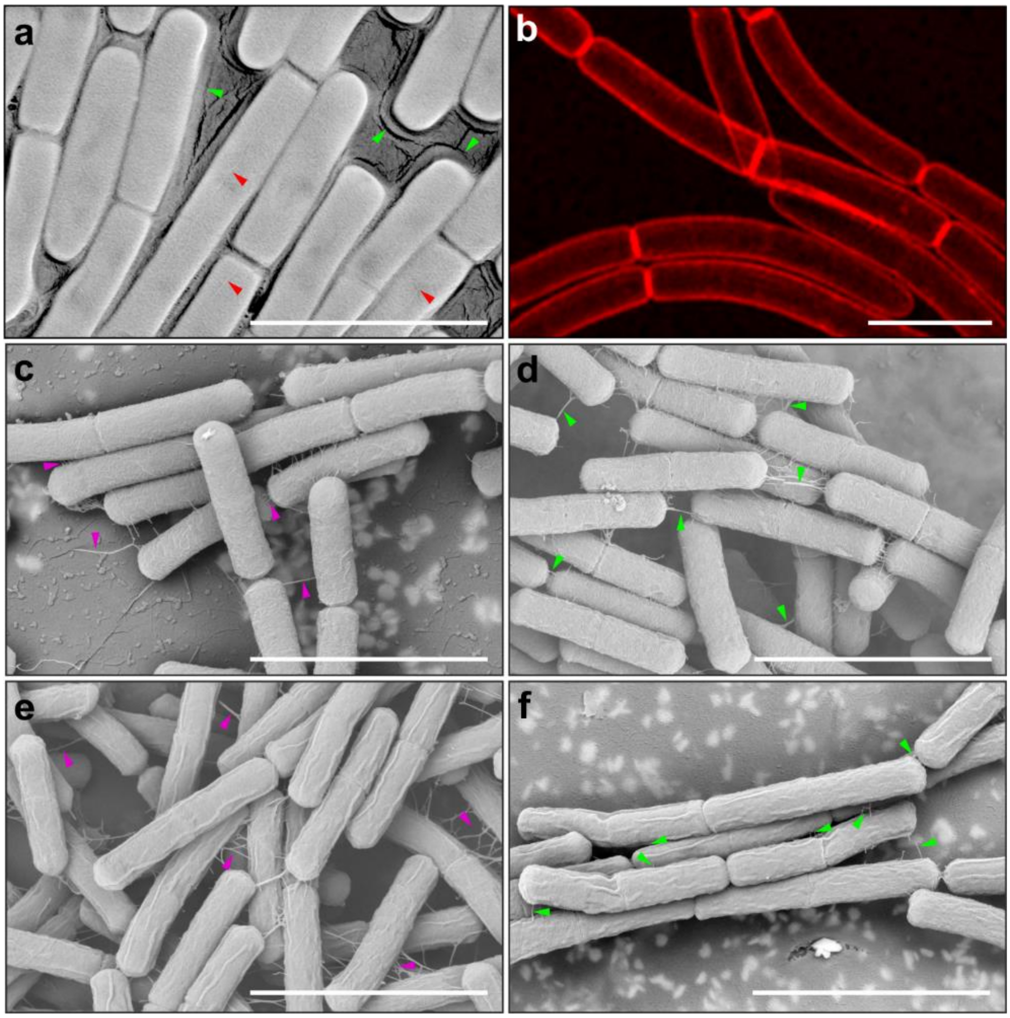

Supplementary Figure 17 | Comparison of various methods for observing bacteria.

(**a**) wt *B. subtilis* (LK1432) grown on LB agar covered with cellophane for 4 hours at 37 °C. The cellophane containing bacteria was carefully removed and fixed and coated for SEM. An intact layer of extracellular matrix (green arrows) and likely flagella (red arrows) are clearly visible. Scale bar=3 µm.

(**b**) wt *B. subtilis* (LK1432) grown on a thin layer of a 1× PBS agarose pad lying atop LB agar containing Nile Red for 4 hours. The 1×PBS agarose pad was covered with a coverslip and observed by SIM. Membranes were stained with Nile Red (red). No membranous structures were observed in the extracellular space. Scale bar=3 µm.

(**c**) wt *B. subtilis* (LK1432) grown in the same way as for SIM analysis. After 4 hours, bacteria were imprinted on EM grids, fixed and coated. SEM showed tubular structures – flagella and possibly filaments made of extracellular matrix (magenta arrows). Scale bar=3 µm.

(**d**) Δ*hag* (LK1966) grown and imaged in the same way as in panel **c**. As the strain lacks flagella, the green arrows show likely filaments made of disrupted extracellular matrix. Scale bar=3 µm.

(**e**) wt *B. subtilis* (LK1432) grown for 4 hours on nitrocellulose membrane-supported EM grids. After 4 hours, bacteria were fixed and coated. As in panel **c**, SEM showed tubular structures – flagella and possibly filaments made of extracellular matrix (magenta arrows). Scale bar=3 µm.

(**f**) Δ*hag* (LK1966) grown and imaged in the same way as in panel **e**. As the strain lacks flagella, the green arrows show likely filaments made of disrupted extracellular matrix. Scale bar=3 µm.

All experiments (panels a-f) were conducted in 3 biological replicates.

Supplementary Table 1 | List of strains used in this study

Experimental Models: Organisms/Strains	Source
LK1432 (*B. subtilis* BSB1 wt-derivative 168 trp$^+$)	[23]
LK1629 (*B. subtilis* PY79 wt-derivative 168 trp$^+$)	[58]
LK1780 (*B. megaterium* wt CCM3380)	Czech Collection of Microorganisms (CCM)
LK1553 (*D. raidodurans* R1 wt ATCC-BAA816)	[59]
LK1133 (*E. coli* wt K12 KW72)	[60]
GP902 (*B. subtilis* 168 trpC2-Δ*hag::tet*)	[61]
LK1966 (LK1432 was transformed with WN1237 gDNA-Δ*hag::tet*)	This study
DS1895 (*B. subtilis* PY79-*amyE::*P*hag-hag*^{T209C}*::spc*)	[19]
LK2052 (LK1432 was transformed with DS1895 gDNA-*amyE::Phag-hag*^{T209C}*::spc*	This study
WN1237 (WN716-Δ*sigD::kan*)	[62]
LK1873 (LK1432 was transformed with WN1237 gDNA-Δ*sigD*::*kan*)	This study
LK1550 (*B. subtilis* BSB1-Δ*sigI* Δ*rsgI::spc*)	[63]
QB5344 (*B. subtilis* 168 trpC2-Δ*sigB::cat*)	J. Stülke
LK2218 (LK1432 was transformed with QB5344 gDNA – Δ*sigB::cat*)	This study
BSU2007 (*B. subtilis* 168 trpC2 Marburg-Δ*sigY* Δ*sigZ* Δ*sigV* Δ*ylaC* Δ*sigX* Δ*sigM* Δ*sigW*)	[64]
1A771 (*B. subtilis* MO1099 trpC2 pheA1- *amyE::ery*)	[65]
LK1922 (LK1432 was transformed with 1A771 gDNA-*amyE::ery*)	This study
LK1925 (LK1432 harboring pCPP31-Y1::*neo, cat*)	This study
LK2317 (LK1432 *amyE::Phyperspank-comK::spc*)	This study
BKE1042 (*B. subtilis* 168 trpC2-Δ*comK::ery*)	[66]
LK2380 (LK1432 was transformed with BKE1042 gDNA-Δ*comK::ery)*	This study
LK1940 (LK1873 Δ*sigD::kan* transformed with 1A771 gDNA-*amyE::ery*)	This study
LK1944 (LK1873 Δ*sigD::kan* harboring pCPP31-Y1::neo, cat)	This study
LK2392 (LK1925 was transformed with BKE1042 gDNA-Δ*comK::ery*)	This study
SB444 (*B. subtilis* PY79-*amyE::*P*hyperspank-gfp::spc*)	[1]
LK2078 (LK1432 was transformed with SB444 gDNA- *amyE::*P*hyperspank-gfp::spc*)	This study
KR318 (*B. subtilis* PY79-*amyE::*P*hyperspank-spoVM*^{P9A}*-gfp::spc*)	[56]
LK2525 (LK1432 was transformed with KR318 gDNA- *amyE::*P*hyperspank-spoVM*^{P9A}*-gfp::spc*)	This study

LK2375 (LK1432 harboring pHY300-P*veg-ZsGreen-term*)	This study
DS1447 (*B. subtilis* 3610- Δ*lytF::tet*)	[67]
LK2125 (LK1432 was transformed with DS1447 gDNA- Δ*lytF::tet*)	This study
BKE09420 (*B. subtilis* 168 trpC2-*ΔlytE::ery*)	[66]
LK2290 (LK2125 Δ*lytF::tet* was transformed with BKE09420 gDNA-Δ*lytE::mls*)	This study
**Recombinant DNA (plasmids)**	
pCPP31-Y1 (Cm^r, Neo^r)	[36]
pHY300 (P*veg*-ZsGreen-term, Tet^r)	[57]
pDR111 (P*hyperspank-comK*, Amp^r, $Spec^r$)	[68]

Supplementary Table 2 | List of primers used in this study.

No.	SEQUENCE (5'-3')	USAGE
**Primers for verification of genes deletion**		
LK1010	GGGAATTCCATATGCAATCCTTGAATTATGAAGATC	verification of Δ*sigD*
LK1011	CCGCTCGAGTCATTATTGTATCACTTTTTCC	verification of Δ*sigD*
LK1004	GGAATTCCATATGACACAACCATCAAAAAC	verification of Δ*sigB*
LK1005	CCGCTCGAGTCATTACATTAACTCCATCGA	verification of Δ*sigB*
LK1164	GGAATTCCATATGGTGAAACCAGTGCTTAGCC	verification of Δ*sigI*
LK1165	CCGCTCGAGTCATGAGTGCAGCACCCC	verification of Δ*sigI*
LK2322	AGCGCTTAACACACTGAACCGTTTGTCTTC	verification of Δ*hag*
LK2323	TTGAAGTACGTTTTGCGGCTGTTGGTTTGC	verification of Δ*hag*
LK2490	GTTACCTCGGTGCGAAAAGATTCTAA	verification of Δ*lytE*
LK2491	ATGAAAAAGCAAATCATTACAGCTACG	verification of Δ*lytE*
LK2680	ATGAAAAAGAAATTAGCAGCAGGG	verification of Δ*lytF*
LK2681	TTAGAAATATCGTTTTGCACCGAG	verification of Δ*lytF*
LK2826	AGTCAGAAAACAGACGCACCTTTAGAATCG	verification of *comK* integration into *amyE* site
LK2827	CTAATACCGTTCCCCGAGCTCACGCAAAAT	verification of *comK* integration into *amyE* site
LK3172	ATGAACGAGAAAAATATAAAACAC	verification of *erm* gene in *amyE* site
LK3173	TTACTTATTAAATAATTTATAGCT	verification of *erm* gene in *amyE* site
**Primers for qPCR: SEQUENCE (5'-3')**		
LK1281	TACGTAATACGACTCACTATAGGGAGACAGCTCGTGTCGTGAGATGT	Preparation of recovery marker
LK1282	CGTTGCTGATCTGCGATTAC	Preparation of recovery marker
LK2910	GAAGTGAACGGCGCAACAAT	*comK* qPCR
LK2911	CTGTCGACAATTTGCAGCGG	*comK* qPCR
LK2618	TCATGTTGCCAGCACGTTAT	recovery marker qPCR
LK2619	AAGGGGCATGATGACTTGAC	recovery marker qPCR

Supplementary Table 3 | Proteins identified by MS analysis as part of the group 'Enriched proteins in Δ*sigD* strain' (t-value ≤ -2).

**Enriched proteins in Δ*sigD* strain**	
**Functional group**	Name
**ABC Transporters**	BmrA, YhaP, MetP, MntA, MntB, YtrE, FtsX, OppA, OppF, RbsC, TcyB, TcyC, YwjA, OpuAA, OpuAC, OpuD, ArtQ, YbaE, YclO, YclP, YclQ, YwrB, YrhG, GltT, CopA, MgtE, GlpF, GlpT, NupC, MleN, PutP, Mdr, AlsT
**ATP synthesis**	AtpB, AtpC, AtpH, AtpE, Pgk, Pyk
**Biosynthesis of cofactors**	NadE, MenH, HemQ
**Lipid metabolism**	PgsA, PlsY
**Biosynthesis/ acquisition of amino acids**	GlnA, CysK, GlyA, CysE
**Biosynthesis/ acquisition of nucleotides**	GuaA, GuaB
**Carbon metabolism**	PfkA, CitB, FbaA, PdhB, PdhC, Eno, Pta, Tkt, Mdh, Pgi
**Cell division**	SepF, RasP, DivIB
**Cell envelope stress proteins (controlled by SigM, V, W, X, Y)**	YteJ, YqfB
**Cell shape**	RodZ, MreC
**Cell wall degradation/ turnover**	GamP, LytE
**Cell wall synthesis**	PgcA, TagG, TagV, MurAA, MurD
**Cell wall/ other**	WalK, YycH
**DNA condensation/ segregation**	Hbs, Soj
**DNA repair/ recombination**	RuvA, PnpA
**Electron transport/ other**	TrxA, Fer
**General stress proteins (controlled by SigB)**	YitT, SodA
**Chaperones/ protein folding**	GrpE, GroES
**Membrane dynamics**	FloT
**Metabolism of signaling nucleotides**	CdaR, GdpP
**Motility and chemotaxis**	YcdA, MotP
**Proteins of unknown function**	YfmQ, YbfF, YrbF, YvbI, YjhA, YxaI, YneF, YpmS, YwnC, YheA, YerA, YukB, YukE, YugP, YhbJ, YlmG, YdjN, YtkA, YqfF, YvfG, YukC
**Phage-related functions**	YueB
**Phosphate metabolism**	PhoR
**Phosphorelay**	YaaT
**Phosphotransferase systems**	NagP, SacP, PtsG, TreP, BglP, FruA
**Protein modification**	Lgt
**Protein secretion**	SecDF, SecG, SecY, SipS, SipT, SpoIIIJ, LspA, YacD
**Regulators of electron transport**	ResE
**Resistance against oxidative and electrophile stress**	Tpx
**Respiration**	QoxB, NarH, QcrB
**RNA chaperones**	CspD

**Regulation of gene expression**	AbrB, Abh, YhcB, RsiX
**Sporulation proteins**	YlbC
**Translation**	GatA, YlaG, Tgt, YtpR, RplC, RplL, RplT, RplU, GltX, TyrS, MetS, IleS, Frr, LysS
**Utilization of amino acids**	AnsB, Bcd, Ald, Pdp
**Utilization of specific carbon sources**	YtsJ, GlpK, RbsK, MleA

Supplementary Table 4 | Proteins identified by MS analysis as part of the group 'Membrane fraction proteins in both wt and Δ*sigD*' (t-value [2,-2]).

**Membrane fraction proteins in both wt and Δ*sigD***	
**ABC Transporters**	ArtP, EcsA, FeuA, FhuC, FhuD, FtsE, MetN, MetQ, MsmX, OppC, OpuAB, PstBA, PstS, RbsA, RbsB, RbsD, SufC, TcyA, YbxA, YckB, YdbJ, YfiY, YfmC, YfmM, YhaQ, YhcH, YhfQ, YknW, YknX, YkpA, YtrB, YtrF, YusV, YvgL, YvrC, YxeB, YybJ, ZnuA, ZnuC
**Electron transport and ATP synthesis**	AckA, AtpA, AtpD, AtpF, AtpG, SucC, Rex, ResD, EtfA, YumC, YojN, YutJ
**Biosynthesis of cofactors**	GsaB, HemB, HemY, HepT, LipA, MenB, PanB, PdxK, PdxS, RibH
**Lipid metabolism**	AccA, AccB, AccC, AccD, AcpA, FabG, FabI, FabL, GpsA, IspG, PlsC, PlsX, Psd
**Biosynthesis/ acquisition of amino acids**	AroA, AsnB, AspB, DapA, DapG, MetK, MtnA, MtnK, ProA, Prs, SerA
**Biosynthesis/ acquisition of nucleotides**	Adk, Dra, Gmk, Ndk, NrdF, PupG, PurA, PyrAB, PyrG, PyrH, Tmk, Udk, Upp
**Carbon metabolism**	CggR, CitG, CitZ, GapA, GapB, GlpX, GndA, Icd, OdhA, OdhB, PckA, PdhA, PdhD, Pgm, PycA, SdhA, SdhB, SdhC, Tpi, YqfL, YwjH, Zwf, AcoA, AcoB, AcoL, AcsA, FruC, GalE, GalK, GlpD, GntZ, IolS, LicH, LutB, MalK, MalR, TreA
**Cell division**	EzrA, FtsA, FtsH, FtsZ, GpsB, MinD, Noc
**Cell envelope stress proteins (controlled by SigM, V, W, X, Y)**	PspA, YaaN, YdbS, YebC, YoaF, YobJ, YpuA
**Cell shape**	Mbl, MreB
**Cell wall degradation/ turnover**	IseA, LytC, LytG
**Cell wall synthesis**	DacA, Ddl, DltA, DltC, DltD, GgaB, GlmM, GlmS, GtaB, LtaS, MurB, MurC, MurG, PatA, PbpA, PbpB, PbpC, PbpD, PbpX, PonA, RacE, TagD, TagE, TagF, TagH, TagT, TagU, YfnI, YkuQ, PdaC, WalR, WapA, WprA, YodJ
**Coping with hypo-osmotic stress**	MscL
**RNA synthesis and degradation**	CshA, CshB, CspB, RnjA, RnjB, Rny, NusA, NusG, Rho, RpoA, RpoB, RpoC, RpoE, SigH, SigW, SigX, CodY, DegU, SalA, YmcA, RsiW, YhdL
**DNA condensation/ segregation**	GyrB, ParE, Spo0J, SpoIIIE
**DNA repair/ recombination**	MutSB, PcrA, PolA, RecA
**DNA replication**	DnaA, DnaI, DnaN, PolC, TopB, YpcP
**DNA restriction/ modification**	YdiS
**General stress proteins (controlled by SigB)**	CsbB, YacL, YceC, YceD, YceE, YceH, YtxG, YtxH, YwsB
**Genetic competence**	BdbD, Med, Nin, Rok, YvcJ
**Chaperones/ protein folding**	DnaJ, DnaK, GroEL, HtpG, PpiB, Tig
**Iron metabolism**	SufD, SufS
**Membrane dynamics**	FloA, FabF
**Miscellaneous metabolic pathways**	SpeE, SrfAA, SrfAB, SrfAC, SrfAD
**Motility and chemotaxis**	FlhA, FlhF, TlpC

**Proteins of unknown function**	DhaS, LepA, MraW, YaaK, YccF, YcnI, YddK, YdeO, YdjG, YeaC, YerC, YerH, YfhC, YgaE, YhaH, YhdP, YheB, YhgE, YjlC, YkaA, YkyA, YkzS, YlbA, YlbL, YloU, YloV, YoxD, YpmB, YqeG, YqeY, YqgA, YqhL, YqhY, YqiW, YqjE, YqzC, YqzD, YrhD, YrhE, YrkA, YrrL, YrvD, YsbA, YshE, YtsP, YubF, YueD, YugI, YutD, YutI, YvbJ, YvrP, YwhC, YwtE, YxiF, YxkD, YxxG, YyaF, YydB
**Phosphate metabolism**	PpaC
**Phosphotransferase systems**	ManP, MtlA, PtsH, PtsI
**Protein modification**	McsB, RsbW
**Protein secretion**	Ffh, FtsY, PrsA, SecA, SppA
**Proteolysis**	ClpC, ClpX, ClpY, CtpA, HtpX, HtrB
**Resistance against oxidative and electrophile stress**	AhpC, KatA
**Resistance against toxins/ antibiotics**	SunI, SwrC, YokG
**Respiration**	CccA, CccB, CtaC, NarG, Ndh, QcrA, QoxA
**SP-beta prophage**	YqaP, YokE, YonB, YonC, YopQ
**Sporulation**	Spo0M
**Sulfur metabolism**	CymR
**Translation**	AlaS, AsnS, AspS, EngA, Era, FolD, FusA, GatB, GidA, GlyS, InfA, InfB, InfC, KsgA, LeuS, Obg, PheT, PrfA, ProS, QueA, QueC, RbfA, RbgA, RplA, RplB, RplD, RplE, RplF, RplJ, RplK, RplM, RplN, RplO, RplP, RplQ, RplR, RplS, RplV, RplW, RplX, RpmA, RpmC, RpmD, RpmI, RpsB, RpsC, RpsD, RpsE, RpsF, RpsG, RpsH, RpsI, RpsJ, RpsK, RpsL, RpsM, RpsN, RpsO, RpsP, RpsR, RpsS, RpsU, SerS, ThdF, ThrS, TrmB, Tsf, TufA, YloN, YmcB, YpfD, YqeH, YqeI, YqeT, YqeV
**Transporters/ other**	DctP, KtrC, LutP, MdtP, MrpG, SteT, YbeC, YclF, YcnJ, YutK, YvsH, YwbM, YwbN, ZosA
**Utilization of amino acids**	AnsA, BkdB, GudB, Kbl, LpdV, RocD, SdaAA, SdaAB, Tdh
