# Supplementary material for: Bacterial nanotubes as a manifestation of cell death
Source: Nat Commun. 2020 Oct 2;11:4963. doi: 10.1038/s41467-020-18800-2 (PMC7532143; doi:10.1038/s41467-020-18800-2)
Supplement: Supplementary file 3 — Description of Additional Supplementary Files [file 41467_2020_18800_MOESM3_ESM.pdf]

Description of Additional Supplementary Files

File Name: Supplementary Movie 1
Description: **Conditions that induce formation of NTs.** A field containing a large number of wt (LK1432) cells. This sample was prepared using the P-GLG method. Time-lapse fluorescence microscopy movie. Pictures were taken at the indicated time points (left upper corner). Left panel-Phase contrast; middle panel-Membrane staining (Nile Red- red); right panel-Merge of Phase contrast and membrane staining.

File Name: Supplementary Movie 2
Description: **The process of dying: the order of events.** A field containing wt (LK1432) cells. This sample was prepared using the P-GLG method. Time-lapse fluorescence microscopy movie. Fig. S5 and its accompanying legend describe the movie.

File Name: Supplementary Movie 3
Description: **Terminal structure of NTs.** Time-lapse microscopy of wt (LK1432) prepared by the P-GLG method. Images were taken each minute. Membranes were stained by Nile Red (red) – middle panel. Left panel – phase contrast, right panel – merge (phase contrast + Nile red).
